# Quantitative and qualitative evaluation of hybrid iterative reconstruction, with and without noise power spectrum models: A phantom study

**DOI:** 10.1002/acm2.12304

**Published:** 2018-02-28

**Authors:** Kazuya Minamishima, Koichi Sugisawa, Yoshitake Yamada, Masahiro Jinzaki

**Affiliations:** ^1^ Office of Radiation Technology Keio University Hospital Shinjuku Japan; ^2^ Department of Radiology Keio University School of Medicine Tokyo Japan

**Keywords:** computed tomography, iterative reconstruction, noise power spectrum model, radiation dose

## Abstract

The purpose of this phantom study was to investigate the feasibility of dose reduction with hybrid iterative reconstruction, with and without a noise power spectrum (NPS) model, using both quantitative and qualitative evaluations. Standard dose (SD), three‐quarter dose (TQD), and half‐dose (HD) of radiation were used. Images were reconstructed with filtered back projection (FBP), adaptive iterative dose reduction 3D (AIDR 3D) (MILD, STR), and AIDR 3D enhanced (eAIDR 3D) (eMILD, eSTR). An NPS analysis, task‐based modulation transfer function (MTF_task_) analysis, and comparisons of low‐contrast detectability and image texture were performed. Although the eAIDR 3D had a higher NPS value in the high‐frequency range and improved image texture and resolution as compared with AIDR 3D at the same radiation dose and iteration levels, it yielded higher noise than AIDR 3D. Additionally, although there was no statistically significant difference between SD‐FBP and the TQD series in the comparison of the mean area under the curve (AUC), the mean AUC was statistically significantly different between SD‐FBP and the HD series. NPS values in the high‐frequency range, 10% MTF_task_ values, low‐contrast detectability, and image textures of TQD‐eMILD were comparable to those of SD‐FBP. Our findings suggested that using eMILD can reduce the radiation dose by 25%, while potentially maintaining diagnostic performance, spatial resolution, and image texture; this could support selecting the appropriate protocol in a clinical setting.

## 1 INTRODUCTION

The increasing use of computed tomography (CT) examinations in clinical settings has resulted in higher levels of patient radiation exposure.^1^ Therefore, optimization of scan and reconstruction parameters is vital for minimizing the associated cancer risk. As a potential solution to this problem, iterative reconstruction (IR) is a technique that has been developed by several vendors as a method that can allow a reduction in radiation dose.^2^, ^3^, ^4^, ^5^, ^6^, ^7^, ^8^ Although these techniques iteratively reduce noise in the image space, raw data, or both, IR techniques have also been reported to produce changes in image texture.^5^, ^9^, ^10^, ^11^, ^12^

Adaptive iterative dose reduction 3D (AIDR 3D) (Toshiba Medical Systems, Otawara, Japan) is a hybrid IR technique that uses a scanner model and statistical noise model, together with projection noise estimation in the raw data domain, to reduce photon and electronic noise.^13^, ^14^ Several previous studies have indicated that AIDR 3D improves image quality and reduces dose in a manner comparable to IR.^15^, ^16^ In contrast, it has also been reported that resolution changes in accordance with radiation dose, iterative strength, and contrast, and that low contrast detectability is not necessarily improved at low dose levels.^17^, ^18^

AIDR 3D Enhanced (eAIDR 3D) is an IR‐mounted noise power spectrum (NPS) model that preserves high‐frequency noise in the NPS and is expected to offer improved image texture and resolution as compared to AIDR 3D. Yet, to the best of our knowledge, no study has yet evaluated the image quality characteristics of eAIDR 3D in detail. Additionally, it is known that quantitative evaluations of IR, such as contrast‐to‐noise ratio analyses, diverge from qualitative evaluations, because IR is a nonlinear reconstruction method.^18^ Therefore, both quantitative and qualitative evaluations are necessary for assessing IR image quality.

The purpose of this study was to investigate the feasibility of dose reduction with hybrid iterative reconstruction, with and without an NPS model, in a phantom using both quantitative and qualitative evaluations.

## 2 MATERIALS AND METHODS

The NPS analysis and task‐based modulation transfer function task (MTF_task_) analysis were performed as quantitative evaluations. Low‐contrast detectability was compared using a receiver operating characteristic (ROC) curve analysis and visual image texture was compared using Scheffe's method of paired comparisons, as qualitative evaluations.

### 2.A Scanning and reconstruction

All images were acquired on a 320‐detector row CT scanner (Aquilion ONE Vision edition, Toshiba Medical Systems, Otawara, Japan). All scans were performed at 120 kVp, 0.5 s/rotation, and under automatic exposure control (AEC) with a noise index of 10 (standard dose [SD]), 12 (three‐quarter dose [TQD], 25% dose reduction), and 15 (half‐dose [HD], 50% dose reduction) for a slice thickness of 5 mm, in the case of abdominal CT.^19^ The volume CT dose indices (CTDIvol) were 3.5, 2.6, and 1.7 mGy for quantitative evaluations and 6.3, 4.7, and 3.1 mGy for qualitative evaluations, respectively. As described below, the phantom that was used for quantitative evaluation was filled with diluted contrast medium. Therefore, AEC for quantitative evaluation indicated a larger dose level than qualitative evaluation, and consequently, two different dose levels were used for qualitative and quantitative evaluations.

For quantitative evaluations, non‐helical scanning with an 80 × 0.5 mm^2^ detector configuration was performed to eliminate the influence of table movement on the image‐averaging process, as described later.^17^ For qualitative evaluation, helical scanning (pitch factor, 0.844) with a 32 × 1.0 mm^2^ detector configuration was used, assuming clinical settings. All images were reconstructed with a display field‐of‐view (DFOV) of 200 mm, a reconstruction kernel of FC03, and a slice thickness of 5 mm (except in the MTF_task_ analysis, in which the slice thickness was 1 mm). The SD image series was reconstructed with filtered back projection (FBP) and the TQD and HD series were reconstructed with four different iteration levels: AIDR 3D mild and strong (MILD and STR) and eAIDR 3D mild and strong (eMILD and eSTR). Thus, a total of nine protocols were evaluated (SD‐FBP, TQD‐MILD, TQD‐STR, TQD‐eMILD, TQD‐eSTR, HD‐MILD, HD‐STR, HD‐eMILD, and HD‐eSTR). All analyses were performed using these nine protocols, with SD‐FBP as the reference standard.

### 2.B Noise power spectrum (quantitative) analysis

**Figure 1:**
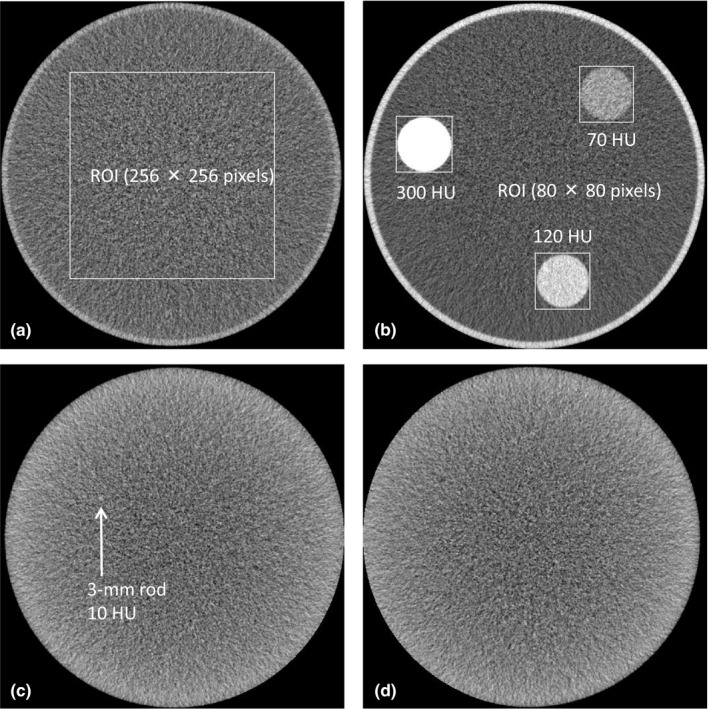
Phantom images used for quantitative and qualitative analyses. (a) Image used for the noise power spectrum (NPS) analysis. The NPS was calculated using the radial frequency method from a region of interest (ROI) and used to calculate the relative noise and peak frequency for each protocol. (b) Image used for the task‐based modulation transfer function (MTF_task_) analysis. The 10% MTF_task_ values were calculated using the radial edge method, from three ROIs, at contrasts of 10, 70, 120, and 300 HU for each protocol. (c) Image used for the comparison of low contrast detectability of a 3‐mm rod at a contrast of 10 HU. Low contrast detectability was assessed using a receiver operating characteristic curve analysis. (d) Image used for comparison of both low contrast detectability and visual image texture. Visual image texture was assessed using Scheffe's method of paired comparisons.

An acrylic phantom with a diameter of 200 mm was filled with water and used for NPS analysis [Fig. 1(a)]. The acrylic phantom was placed at the isocentre of the CT scanner. To acquire NPS for each reconstructed image, a region of interest (ROI) of 100 cm^2^ (256 × 256 pixels) was placed on the centre of the image, as shown in Fig. 1(a). NPS was calculated by the radial frequency method (CT measure version 0.97b, Japanese Society of CT Technology, Hiroshima, Japan).^20^, ^21^, ^22^, ^23^ To improve the accuracy of NPS data, 10 scans were performed with the same table position for each radiation dose level, and a total of 10 NPS curves were averaged for each protocol. The integral values of these NPS curves were used to calculate the relative noise value for each protocol, normalized by that of SD‐FBP. Additionally, the Steel‐Dwass test of relative noise was performed for each protocol. The significance level for all evaluations was 5%. Furthermore, normalized NPS (nNPS) curves were calculated by dividing the NPS value by the integral values of these NPS curves.^24^

### 2.C Modulation transfer function_task_ (quantitative) analysis

An acrylic phantom with a diameter of 200 mm, containing three objects with diameters of 30 mm, including soft tissue (70 Hounsfield units [HU]), acrylic (120 HU), and polyoxymethylene (300 HU) was filled with water and used for the MTF_task_ analysis [Fig. 1(b)]. The acrylic phantom was placed at the isocenter of the CT scanner. To acquire low‐noise images for the MTF_task_ analysis, 100 or more scans were performed with the same table position for each radiation dose level and these were averaged for each protocol using image‐averaging techniques.^17^ Furthermore, images were reacquired after the liquid in the phantom was adjusted with diluted contrast medium (60 HU) to obtain a contrast of 10 HU between background and soft tissue (70 HU).

To acquire MTF_task_ values for each averaged image, an ROI of 9.77 cm^2^ (80 × 80 pixels) was placed around the three objects as shown in Fig. 1(b). MTF_task_ values were calculated using the radial edge method, with contrasts of 10, 70, 120, and 300 HU, using software (CT measure version 0.97b).^22^, ^23^, ^25^ The MTF_task_ analyses were used to calculate 10% MTF_task_ values for each protocol.

### 2.D Comparison of low contrast detectability using a ROC (qualitative) analysis

An acrylic phantom with a diameter of 200 mm containing a 3‐mm diameter acrylic bar (120 HU) was filled with dilute contrast medium adjusted to 110 HU to obtain a contrast of 10 HU between background and the acrylic bar and used to assess low contrast detectability [Fig. 1(c)]. The phantom assumes a small size and low‐contrast liver lesion, and the size and contrast of the lesion was determined with reference to the previous studies.^26^, ^27^ The phantom was placed at the isocenter of the CT scanner. An ROC analysis was performed to assess low‐contrast detectability. Each protocol included 60 images: 30 with the acrylic bar inserted and randomly positioned (positive images) [Fig. 1(c)] and 30 without the acrylic bar (negative images) [Fig. 1(d)]. Each of the 60 images was displayed in a randomized order on the monitor and analyzed with a continuously distributed test. DBM MRMC software (Department of Radiology, University of Chicago, Chicago, IL, USA) was used to calculate the mean area under curve (AUC) and 95% confidence intervals for each protocol, and to calculate the difference in the average AUC and *P*‐values between each protocol.^28^, ^29^, ^30^, ^31^, ^32^, ^33^ The significance level for all evaluations was 5%. Because this software employs the jackknife method and has been used in a number of previous studies, both between‐case and between‐reader variations can be considered.^28^, ^29^, ^30^, ^31^, ^32^, ^34^, ^35^, ^36^ The ROC analysis was performed by five radiological technologists (3–20 yr’ experience) on a 1 M liquid crystal display (RadiForce RS110, EIZO, Ishikawa, Japan) with a window width and level of 130 and 100 HU, respectively. The observation time and distance were arbitrary. Consent for the publication of the results was obtained from the observers.

### 2.E Comparison of visual image texture using Scheffe's method of paired comparisons (qualitative)

Negative images obtained in ROC analyses were used to assess image texture [Fig. 1(d)]. A total of 36 pairs selected from the nine protocols were displayed side‐by‐side on a monitor and scored on a 5‐point scale (+2: left image was definitely better; +1: left image was slightly better; 0: images were equal; −1: left image was slightly worse; −2: left image was definitely worse). Image texture was compared using Scheffe's method of paired comparisons (Nakaya's modified model).^23^, ^37^, ^38^, ^39^ The difference in the mean preference scale between each protocol was calculated with Microsoft Excel 2013 (Microsoft, Redmond, WA, USA). The significance level for all evaluations was 5%. Image texture was compared by five radiological technologists (3–20 yr’ experience) on a 1 M liquid crystal display (RadiForce RS110, EIZO, Ishikawa, Japan) with a window width and level of 130 and 100 HU, respectively. The observation time and distance were arbitrary. Consent for the publication of the results was obtained from the observers.

## 3 RESULTS

### 3.A Noise power spectrum analysis

**Figure 2:**
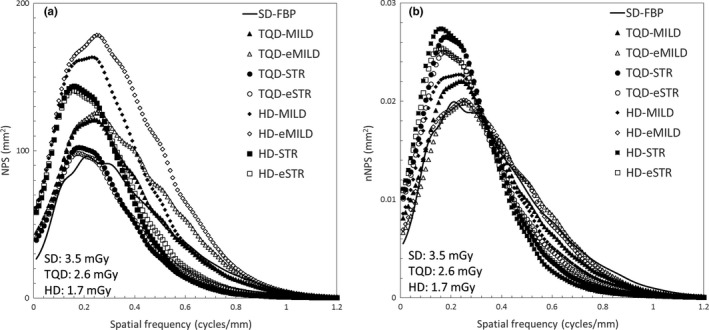
Noise power spectrum (NPS) curves (a) and normalized NPS (nNPS) curves (b) for standard dose with filtered back projection (SD‐FBP), three‐quarter dose (TQD), and half‐dose (HD) with adaptive iterative dose reduction 3D (AIDR 3D) (TQD‐MILD, TQD‐STR, HD‐MILD, and HD‐STR) and AIDR 3D enhanced (TQD‐eMILD, TQD‐eSTR, HD‐eMILD, and HD‐eSTR).

Figure 2 shows NPS curves (a) and nNPS (b) curves. The nNPS curves revealed that TQO‐MILD, TQD‐STR, TQD‐eSTR, HD‐MILD HD‐STR, and HD‐eSTR tended to have high NPS values in the low frequency and low NPS values in the high frequency, as compared with SD‐FBP. However, the nNPS curves of TQD‐eMILD and HD‐eMILD were similar to that of SD‐FBP. Furthermore, the eAIDR 3D had a lower NPS value in the low frequency range and had a higher NPS value in the high frequency range than the AIDR 3D, at the same radiation dose and iteration level. The relative noise value of eAIDR 3D was higher than that of AIDR 3D at the same radiation dose level and iteration level (Tables 1 and 2).

**Table 1:** Relative noise for each protocol

Protocol	Relative noise
SD‐FBP	1.00 ± 0.024
TQD‐MILD	1.14 ± 0.047
TQD‐STR	0.80 ± 0.030
TQD‐eMILD	1.31 ± 0.043
TQD‐eSTR	0.81 ± 0.032
HD‐MILD	1.49 ± 0.058
HD‐STR	1.09 ± 0.052
HD‐eMILD	1.84 ± 0.055
HD‐eSTR	1.15 ± 0.051

eMILD, adaptive iterative dose reduction 3D enhanced mild; eSTR, adaptive iterative dose reduction 3D enhanced strong; FBP, filtered back projection; HD, half‐dose; MILD, adaptive iterative dose reduction 3D mild; SD, standard dose; STR, adaptive iterative dose reduction 3D strong; TQD, three‐quarter dose.

**Table 2:** Steel‐Dwass test of relative noise for each protocol

	Difference	*P*‐values
TQD‐MILD—SD‐FBP	0.14	<0.001
SD‐FBP—TQD‐STR	0.20	<0.001
TQD‐eMILD—SD‐FBP	0.31	<0.001
SD‐FBP—TQD‐eSTR	0.19	<0.001
HD‐MILD—SD‐FBP	0.49	<0.001
HD‐STR—SD‐FBP	0.09	<0.001
HD‐eMILD—SD‐FBP	0.84	<0.001
HD‐eSTR—SD‐FBP	0.15	<0.001
TQD‐eMILD—TQD‐MILD	0.17	<0.001
TQD‐eSTR—TQD‐STR	0.01	<0.001
HD‐eMILD—HD‐MILD	0.35	<0.001
HD‐eSTR—HD‐STR	0.06	<0.001

eMILD, adaptive iterative dose reduction 3D enhanced mild; eSTR, adaptive iterative dose reduction 3D enhanced strong; FBP, filtered back projection; HD, half‐dose; MILD, adaptive iterative dose reduction 3D mild; SD, standard dose; STR, adaptive iterative dose reduction 3D strong; TQD, three‐quarter dose.

### 3.B Modulation transfer function_task_ analysis

**Figure 3:**
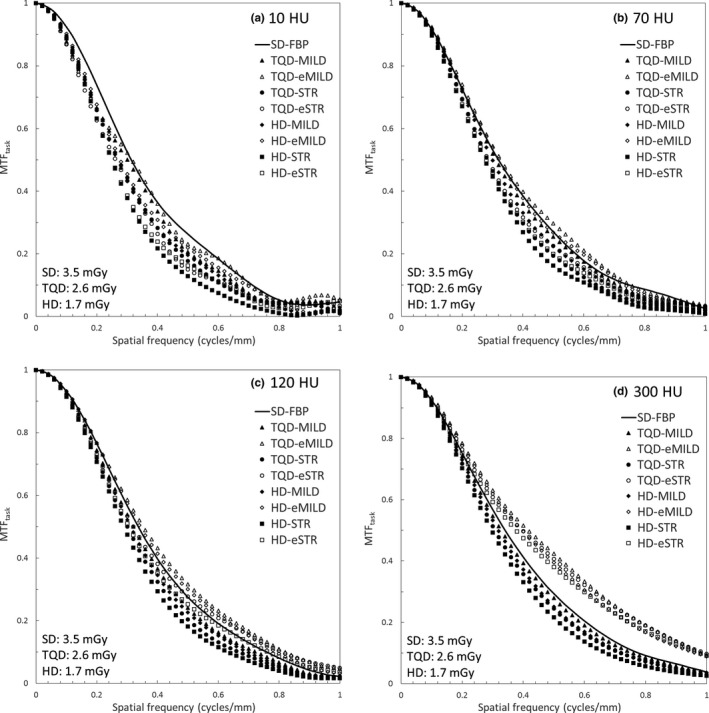
Task‐based modulation transfer function (MTF_task_) curves for standard dose with filtered back projection (SD‐FBP), three‐quarter dose (TQD), and half‐dose (HD) with adaptive iterative dose reduction 3D (AIDR 3D) (TQD‐MILD, TQD‐STR, HD‐MILD, and HD‐STR) and AIDR 3D enhanced (TQD‐eMILD, TQD‐eSTR, HD‐eMILD, and HD‐eSTR) at contrasts of (a) 10 HU, (b) 70 HU, (c) 120 HU, and (d) 300 HU for each protocol.

Table 3 and Fig. 3 show the 10% MTF_task_ values and MTF_task_ curves. Although both AIDR 3D and eAIDR 3D had lower 10% MTF_task_ values with lower CT values, lower radiation doses, and higher iteration levels, the 10% MTF_task_ values were higher for eAIDR 3D than for AIDR 3D at the same radiation doses and iteration levels. The 10% MTF_task_ values of all AIDR 3D protocols were equal to or lower than those of SD‐FBP. In contrast, the 10% MTF_task_ values of eAIDR 3D at TQD and HD tended to be higher at 120 and 300 HU, and tended to be equal to or lower than those of SD‐FBP at 10 and 70 HU. The 10% MTF_task_ values of TQD‐eMILD at 10 and 70 HU were equal to those of SD‐FBP.

**Table 3:** 10% MTF_task_ values for each protocol and contrast

Protocols	10 HU	70 HU	120 HU	300 HU
SD‐FBP	0.71	0.75	0.76	0.78
TQD‐MILD	0.67	0.70	0.72	0.76
TQD‐STR	0.62	0.64	0.67	0.72
TQD‐eMILD	0.71	0.75	0.82	0.99
TQD‐eSTR	0.63	0.70	0.81	0.99
HD‐MILD	0.65	0.69	0.70	0.72
HD‐STR	0.55	0.62	0.63	0.67
HD‐eMILD	0.69	0.74	0.79	0.97
HD‐eSTR	0.62	0.68	0.76	0.98

eMILD, adaptive iterative dose reduction 3D enhanced mild; eSTR, adaptive iterative dose reduction 3D enhanced strong; FBP, filtered back projection; HD, half‐dose; MILD, adaptive iterative dose reduction 3D mild; MTF, task‐based modulation transfer function; SD, standard dose; STR, adaptive iterative dose reduction 3D strong; TQD, three‐quarter dose.

### 3.C Comparison of low contrast detectability using an ROC analysis

Tables 4 and 5 show the results of the ROC analysis. There was no statistically significant difference in the mean AUC value between SD‐FBP and the series of TQD; however, there was statistically significant difference in mean AUC value between SD‐FBP and the series of HD. Although the mean AUCs of TQD‐eMILD and TQD‐eSTR were significantly higher than those of TQD‐MILD and TQD‐STR, the mean AUCs of HD‐eMILD and HD‐eSTR were significantly lower than those of HD‐MILD and HD‐STR.

**Table 4:** Average AUC and 95% CI for each protocol

Protocols	Average AUC	95% CI
SD‐FBP	0.817	0.742, 0.893
TQD‐MILD	0.709	0.620, 0.799
TQD‐STR	0.773	0.692, 0.854
TQD‐eMILD	0.746	0.657, 0.834
TQD‐eSTR	0.809	0.715, 0.903
HD‐MILD	0.671	0.580, 0.763
HD‐STR	0.652	0.569, 0.734
HD‐eMILD	0.626	0.545, 0.706
HD‐eSTR	0.592	0.503, 0.682

AUC, area under the curve; CI, confidence interval; eMILD, adaptive iterative dose reduction 3D enhanced mild; eSTR, adaptive iterative dose reduction 3D enhanced strong; FBP, filtered back projection; HD, half‐dose; MILD, adaptive iterative dose reduction 3D mild; SD, standard dose; STR, adaptive iterative dose reduction 3D strong; TQD, three‐quarter dose.

**Table 5:** Difference of average AUC, 95% CI, and *P*‐values between each protocol

	Interval AUC	95% CI	*P*‐values
SD‐FBP—TQD‐MILD	0.108	−0.006, 0.222	0.062
SD‐FBP—TQD‐STR	0.045	−0.064, 0.153	0.421
SD‐FBP—TQD‐eMILD	0.072	−0.043, 0.187	0.220
SD‐FBP—TQD‐eSTR	0.008	−0.106, 0.123	0.889
SD‐FBP—HD‐MILD	0.146	0.038, 0.254	0.008
SD‐FBP—HD‐STR	0.166	0.060, 0.272	0.002
SD‐FBP—HD‐eMILD	0.192	0.096, 0.287	<0.001
SD‐FBP—HD‐eSTR	0.225	0.116, 0.334	<0.001
TQD‐eMILD—TQD‐MILD	0.036	0.008, 0.064	0.012
TQD‐eSTR—TQD‐STR	0.037	0.013, 0.060	0.007
HD‐MILD—HD‐eMILD	0.046	−0.005, 0.097	0.075
HD‐STR—HD‐eSTR	0.059	0.002, 0.116	0.041

AUC, area under the curve; CI, confidence interval; eMILD, adaptive iterative dose reduction 3D enhanced mild; eSTR, adaptive iterative dose reduction 3D enhanced strong; FBP, filtered back projection; HD, half‐dose; MILD, adaptive iterative dose reduction 3D mild; SD, standard dose; STR, adaptive iterative dose reduction 3D strong; TQD, three‐quarter dose.

### 3.D Comparison of visual image texture using Scheffe's method of paired comparisons

**Figure 4:**
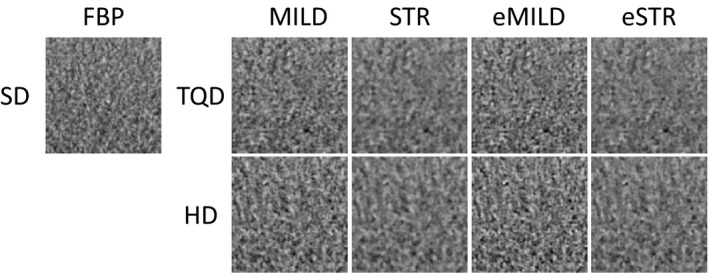
Image texture comparisons. Images assessed by Scheffe's method of paired comparisons included standard dose with filtered back projection (SD‐FBP), three‐quarter dose (TQD), and half‐dose (HD) with adaptive iterative dose reduction 3D (AIDR 3D) (TQD‐MILD, TQD‐STR, HD‐MILD, and HD‐STR) and AIDR 3D enhanced (TQD‐eMILD, TQD‐eSTR, HD‐eMILD, and HD‐eSTR).

**Figure 5:**
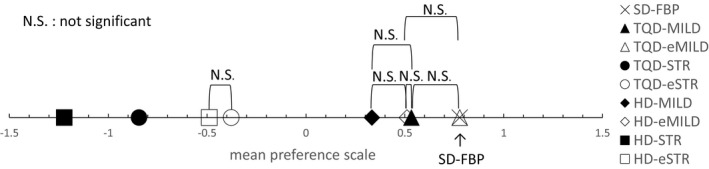
Results of image texture comparisons. Mean preference scale scores for standard dose with filtered back projection (SD‐FBP), three‐quarter dose (TQD), and half‐dose (HD) with adaptive iterative dose reduction 3D (AIDR 3D) (TQD‐MILD, TQD‐STR, HD‐MILD, and HD‐STR) and AIDR 3D enhanced (TQD‐eMILD, TQD‐eSTR, HD‐eMILD, and HD‐eSTR). Results indicate parameter settings that produced image textures similar to that of SD‐FBP. N.S., not significant.

There was no significant difference in the mean preference scale scores among SD‐FBP, TQD‐MILD, TQD‐eMILD, and HD‐eMILD (Figs. 4 and 5). Image texture deteriorated with lower radiation doses and higher iteration levels.

## 4 DISCUSSION

Our study demonstrated that eMILD allowed a 25% reduction in radiation dose while maintaining diagnostic performance, spatial resolution, and image texture. Image quality has not previously been compared between AIDR 3D and eAIDR 3D using both quantitative and qualitative evaluations. We found that NPS values in the high frequency range, 10% MTF_task_ values, low‐contrast detectability, and image texture of TQD‐eAIDR 3D were superior to those of TQD‐AIDR 3D, and similar to those of SD‐FBP. These findings are important because they can guide protocol selection in a clinical setting.

Solomon et al. reported that it is possible to use an NPS to compare image texture quantitatively.^40^ Our NPS analysis findings that the nNPS curves of TQD‐eMILD and HD‐eMILD were close to that of SD‐FBP indicated that the image texture of TQD‐eMILD and HD‐eMILD is similar to that of SD‐FBP.

Our findings that 10% MTF_task_ values changed in accordance with contrast are consistent with a previous study by Richard et al., and represent a feature of nonlinear processing in IR.^25^ We found that although eAIDR 3D improved 10% MTF_task_ values at 120 HU or higher, it did not improve 10% MTF_task_ values at 70 HU or less. Therefore, in clinical settings, eAIDR 3D may be useful for enhanced CT or CT angiography (i.e., imaging with high contrast levels).

We found that the mean AUC value for low‐contrast detectability of TQD‐eMILD was significantly higher than that of TQD‐MILD and was comparable to that of SD‐FBP, and that image texture in TQD‐eMILD was similar to that in SD‐FBP. Thus, we suggest that TQD‐eMILD is desirable for maintaining both diagnostic performance and image texture, while reducing the dose of radiation required. In contrast, the mean AUCs for the low‐contrast detectability values of HD‐MILD, HD‐STR, HD‐eMILD, and HD‐eSTR were significantly lower than that of SD‐FBP, such that a 50% reduction in radiation dose by AIDR 3D or eAIDR 3D may not be feasible for the detection of small low‐contrast lesions.

This study had several limitations. First, we used a uniform water phantom that does not adequately represent the human body (e.g., bones and organs). Furthermore, a phantom diameter of 200 mm does not simulate the size of a human body. Further studies using an anthropomorphic body phantom with embedded low contrast lesions would be required for confirming our preliminary findings. Second, in our qualitative evaluation, images were displayed on monitor at a fixed window level and width. These fixed conditions may have affected the qualitative evaluation. Third, the ROC analysis was only performed at a contrast of 10 HU. In future, more comprehensive assessments should be performed to confirm the clinical applicability of our present findings. Fourth, our ROC curve analysis was performed by five readers with 30 positive and 30 negative images for each protocol, using DBM MRMC software. In an earlier study, it was reported that the software used in the current study requires at least five readers and 25 positive and 25 negative images, to acquire more reliable ROC curves.^33^ Therefore, our statistical analysis of the ROC curve was likely reliable. Nevertheless, to clarify whether the difference in AUC is meaningful or not in terms of detectability, further evaluation with more readers and more images may be necessary.

## 5 CONCLUSION

We suggest that the use of eMILD can facilitate a 25% reduction in radiation dose while potentially maintaining diagnostic performance, spatial resolution, and image texture.

## CONFLICTS OF INTEREST

Masahiro Jinzaki received a research grant from Toshiba Medical Systems. The remaining authors have no financial disclosures to make in relation to this study.

## SOURCES OF FUNDING

This research did not receive any specific grant from funding agencies in the public, commercial, or not‐for‐profit sectors.
